# Immune-Mediated Skin Diseases: Future Therapeutic Perspectives

**DOI:** 10.3390/medicina59101787

**Published:** 2023-10-08

**Authors:** Mauro Alaibac

**Affiliations:** Dermatology Unit, Department of Medicine (DIMED), University of Padua, 35121 Padova, Italy; mauro.alaibac@unipd.it

Immuno-mediated skin diseases are a common and clinically heterogeneous group of cutaneous conditions. The traditional treatment armamentarium of dermatologists, based on broad-spectrum immune modulators, has been revolutionized by the introduction of agents with high specificity, arising from monoclonal and molecular biotechnology and, more recently, from highly targeted small molecule synthesis [1]. Parallel to these therapeutic achievements, our pathogenic understanding of these disorders has greatly advanced in recent years; however, their management is often still challenging owing to their complex pathogenesis and diverse range of clinical manifestations [2].

The introduction of dupilumab, a monoclonal antibody targeting the cytokines IL4 and IL13, which are key players in type 2 inflammatory pathways, recently advanced the treatment paradigm of atopic dermatitis. Beyond the favorable response of skin signs and disease symptoms to dupilumab treatment, which was demonstrated by the pivotal clinical trials, the effect of this drug on the overall quality of life of patients with atopic dermatitis is being extensively studied in real-world populations. In this regard, the genital region is a neglected anatomic site that may be concerned by atopic dermatitis in a percentage ranging from 10 to 45% of cases, where it causes a heavy burden on patients’ psychosocial wellbeing. An interesting prospective study conducted by Napolitano et al. investigated the impact of disease and of its treatment with dupilumab on sexual desire in subjects with atopic dermatitis. Using the validated Sexual Desire Inventory-2 (SDI-2) instrument and a non-validated questionnaire devised by the authors, a significant improvement was demonstrated after 4 and 16 weeks of dupilumab treatment. With this paper, the authors successfully draw attention to an aspect of atopic dermatitis that is commonly overlooked in the dermatological setting and that must be carefully addressed in order to improve patients’ lives [3]. 

Targeting the Janus kinase pathway is a current area of interest in dermatology, where small molecular Janus kinase inhibitors constitute a recent successful addition to the treatment of atopic dermatitis, with the advantage of oral administration compared to injectable monoclonal antibodies. The Janus kinase–signal transducer and activator of transcription is a central proinflammatory and proliferative pathway in the pathogenesis of immune-mediated conditions affecting the skin, such as atopic dermatitis, and other organs. Grieco and al. report on a challenging case of atopic dermatitis unresponsive to topical and systemic treatments, including dupilumab, in a subject suffering from concurrent ulcerative colitis, which was also poorly managed. The initiation of JAK1-selective blocker upadacitinib, at the daily dosage of 15 mg, led to the remission of both atopic dermatitis and ulcerative colitis supporting that this drug may be suitable in the management of coexisting severe conditions mediated by both type 1 and type 2 inflammatory pathways. The authors also suggest that future approaches will take into account targeting the microbiome, considering that cutaneous dysbiosis is a novel contributor to the pathogenesis of atopic dermatitis [4].

In rheumatology, the constant introduction of innovative targeted agents is greatly advancing disease management toward precision medicine and tailored approaches. Systemic sclerosis is an autoimmune connective tissue disorder with distinctive cutaneous findings and internal organ impairment, which is potentially life threatening, with pulmonary complications being the main source of mortality. The treatment of systemic sclerosis has shifted from interventions aimed at symptomatic control to potentially disease-modifying approaches. Fernández-Lázaro et al. systematically reviewed the available evidence of the effect of biological drugs used in this setting. Their analysis included data from 426 patients enrolled in six clinical trials, with a controlled design, on tocilizumab, belimumab, riociguat, abatacept, and romilkimab. Cutaneous involvement, measured using skin disease modified Rodnan scale value (mRSS), demonstrated improvement following treatment with tocilizumab, an anti-IL6 monoclonal antibody; belimumab, an inhibitor of B-lymphocyte stimulator (BLyS) protein; riociguat, an oral small molecule stimulator of soluble guanylyl cyclase; abatacept, a fusion protein between CTLA-4 extracellular domain and IgG1 Fc region; and romilikimab, a bispecific antibody against IL4 and IL13. The latter drug was also shown to stimulate production of non-fibrotic tissue in patients with systemic sclerosis; however, it was withdrawn in 2021. The results of this review support that biological drugs are highly effective in countering the progression of symptoms and fibrosis associated with systemic sclerosis while being more tolerable compared to traditional drugs. Moreover, the introduction of combination regimens with two biologics, such as belimumab and anti-CD20 rituximab, may produce synergistic effects and further improve beneficial outcomes with anti-fibrotic activity [5].

Among biologic agents, monoclonal antibodies targeting the tumor necrosis factor are established in the management of chronic immune conditions in dermatology as well as in rheumatology. Owing to their potent and specific blockade of key pathogenic molecules in the inflammatory cascade and to their high tolerability, the use of tumor necrosis factor inhibitors has been attempted in uncommon disorders that are current areas of unmet therapeutic need. Owczarczyk-Saczonek et al. report on an educational case of aseptic abscess syndrome, a rare condition that is found on the spectrum of neutrophilic dermatoses. This syndrome features sterile abscesses that may severely involve the skin and systemic symptoms. It is frequently associated with a concomitant immune disorder, such as rheumatoid arthritis in the reported patient but may be an isolated finding in more than half of cases. Rapid clinical recognition and an accurate diagnostic workup to exclude differential etiologies is required and allows the initiation of systemic steroid therapy, which is highly effective in controlling the inflammatory symptoms. However, the association with a steroid-sparing immunomodulator is suggested, considering that longer-term treatments may be required: adalimumab therapy was added in this case at a dose of 80 mg subcutaneously followed by 40 mg every two weeks. Finally, the authors reported that adalimumab therapy was continued even when the patient received remdesivir, following symptomatic SARS-CoV-2 infection without complications [6].

In complex and multifactorial disorders, an in-depth pathogenic understanding at the molecular level may aid the development of innovative therapeutic approaches and support their association with traditional remedies, such as herbal preparations. This may be the case of vitiligo, a chronic cutaneous condition that is associated with severe cosmetic impairment and for which the usual therapeutic options provide only limited benefit. It is currently accepted that oxidative stress plays a role in the pathogenesis of vitiligo, where melanocytes may become abnormally susceptible to the damage of reactive oxygen species. Alshaikh and Bharti present an interesting case of stable, long-standing non-segmental vitiligo undergoing clinical improvement following daily supplementation with *Curcuma longa* herbal extracts. The active ingredient curcumin is recognized for its antioxidant properties and the authors propose that it may be incorporated in the current treatment armamentarium for vitiligo as a safe alternative (or adjunctive) approach. Although further evidence from high-quality studies is required, this is an interesting perspective which may also be applicable to vitiligo-like leukoderma occurring in subjects with advanced melanoma undergoing anti-PD1 immunotherapy. Vitiligo occurring in this setting has been associated with an anti-tumor immune response and to increased survival in patients with metastatic melanoma. However, specific treatment may be requested since the cosmetic impairment is associated with a severe worsening in the quality of life: curcumin-based treatment could be evaluated here as a safe and useful approach. Moreover, the onset of vitiligo is also a rarely reported adverse event in patients with psoriasis treated with monoclonal antibodies against IL17, such as ixekizumab, but further insight into the mechanisms underlying this potential side effect is required to support this peculiar association [7].

Cancer immunology is an attractive field for drug discovery, especially in immune-responsive malignancies such as melanoma. Current oncologic immunotherapy for advanced melanoma relies on inhibitors of the PD1 immune checkpoint but their effect is unsatisfactory in up to 50% of cases, resulting in an urgent need to develop novel approaches. Neutrophils are emerging players in cancer biology and have been demonstrated to negatively affect prognosis and therapeutic response in melanoma. A review by our group summarized current knowledge in this filed and proposed future investigations of granulocyte and monocyte apheresis, a non-pharmacologic neutrophil-depleting technique, which could prove safe and effective in combination with checkpoint blockade, especially in those patients with advanced neoplasms that do not respond to cancer immunotherapy alone [8].

In conclusion, this Special Issue focused on recent immunotherapy approaches and novel targeted therapy using bioactive small molecules for the treatment of common and debilitating immune-mediated skin diseases, such as atopic dermatitis. Moreover, the use of biologic agents for the management of rheumatologic disorders with severe cutaneous manifestations has been evaluated, supporting their role as disease modifying agents. In addition, alternative and adjunctive approaches based on herbal preparations with known antioxidant properties have been explored in the treatment of vitiligo, which may also occur as an adverse event in the course of biological therapy and oncologic immunotherapy. Finally, cancer immunology has been reviewed, focusing on the role of tumor-associated neutrophils in melanoma to propose a role for neutrophil-depleting interventions in the management of advanced tumors.

## Data Availability

No new data were created or analyzed in this study. Data sharing is not applicable to this article.
